# Neurobiology of axonal transport defects in motor neuron diseases: Opportunities for translational research?

**DOI:** 10.1016/j.nbd.2017.02.004

**Published:** 2017-09

**Authors:** Kurt J. De Vos, Majid Hafezparast

**Affiliations:** a Sheffield Institute for Translational Neuroscience, Department of Neuroscience, University of Sheffield, Sheffield S10 2HQ, UK; b Neuroscience, School of Life Sciences, University of Sussex, Falmer, Brighton BN1 9QG, UK

**Keywords:** Motor neuron disease, Amyotrophic lateral sclerosis, Axonal transport, Microtubules, Molecular motors, Mitochondria, Neurodegeneration

## Abstract

Intracellular trafficking of cargoes is an essential process to maintain the structure and function of all mammalian cell types, but especially of neurons because of their extreme axon/dendrite polarisation. Axonal transport mediates the movement of cargoes such as proteins, mRNA, lipids, membrane-bound vesicles and organelles that are mostly synthesised in the cell body and in doing so is responsible for their correct spatiotemporal distribution in the axon, for example at specialised sites such as nodes of Ranvier and synaptic terminals. In addition, axonal transport maintains the essential long-distance communication between the cell body and synaptic terminals that allows neurons to react to their surroundings via trafficking of for example signalling endosomes.

Axonal transport defects are a common observation in a variety of neurodegenerative diseases, and mutations in components of the axonal transport machinery have unequivocally shown that impaired axonal transport can cause neurodegeneration (reviewed in El-Kadi et al., 2007, De Vos et al., 2008; Millecamps and Julien, 2013). Here we review our current understanding of axonal transport defects and the role they play in motor neuron diseases (MNDs) with a specific focus on the most common form of MND, amyotrophic lateral sclerosis (ALS).

## 1 Microtubule-based axonal transport

Traditionally two main classes of axonal transport are distinguished based on the overall speed of movement, namely fast axonal transport (~ 50–400 mm/day or 0.6–5 μm/s) and slow axonal transport (0.2–10 mm/day or 0.0002–0.1 μm/s). Slow axonal transport is further subdivided into slow component a (SCa) and b (SCb) based on the proteins transported and the speed, 0.2–3 and 2–10 mm/day, respectively. We now know that both fast and slow axonal transport is mediated by the same molecular motors that move cargoes along microtubules, with the differences in overall speed caused by prolonged pauses between movement phases in slow axonal transport (reviewed in Black, 2016).

Microtubules are polymers made up of tubulin which itself is a heterodimer of α-tubulin and β-tubulin. Microtubules are rigid hollow rods of approximately 25 nm in diameter built from 13 linear protofilaments composed of alternating tubulin heterodimers and arranged around a hollow core. Due to the head to tail arrangement of the tubulin heterodimers microtubules are polarised with a fast growing plus end and a slow growing minus end. The polarity of microtubules dictates the direction of movement of the molecular motors along them.

There are two major families of microtubule based molecular motors, namely the kinesin family which move mostly toward the plus end of microtubules and the cytoplasmic dyneins that move toward the minus end (reviewed in Hirokawa et al., 2010). Because axonal microtubules are uniformly orientated with their plus end pointing away from the cell body (Baas et al., 1988) kinesins mediate anterograde transport away from the cell body toward the axon terminal and cytoplasmic dynein drives retrograde transport from the distal axon toward the cell body.

The human kinesin superfamily contains 45 members, subdivided into 15 subfamilies. The main kinesin family members involved in fast axonal transport are kinesin-1 (previously referred to as conventional kinesin or KIF5), and the kinesin-3 family members KIF1A, KIF1Bα and KIF1Bβ. Anterograde slow axonal transport appears to be mainly mediated by kinesin-1 (Xia et al., 2003). Kinesin-1 is a heterotetramer consisting of two kinesin heavy chains (KHCs) and two kinesin light chains (KLCs). KHC contains the catalytic motor domain, a neck linker region, an α-helical stalk interrupted by two hinge regions, and the tail. The motor domain binds microtubules and hydrolyses ATP to generate force. Together with the neck region, the motor domain conveys processivity and direction of movement. The stalk is required for dimerisation and the tail, together with KLC is involved in regulation of motor activity as well as cargo binding (reviewed in Hirokawa et al., 2010). The latter also involves various adapter proteins such as c-Jun N-terminal kinase (JNK)-interacting protein (JIP) 1, 3 and 4, mitochondrial Rho GTPase (Miro) 1 and 2, trafficking kinesin (TRAK) 1 and 2, and huntingtin that link kinesin-1 to specific cargo, directly or via KLCs (reviewed in Fu and Holzbaur, 2014). In contrast to kinesin-1, KIF1A and KIF1Bα/β are monomeric kinesin motors consisting of an N-terminal motor domain, a conserved stalk domain and a C-terminal pleckstrin homology (PH) that aids in the interaction with cargoes in conjunction with adapter proteins such as DENN/MADD (Differentially Expressed In Normal And Neoplastic Cells/MAP Kinase Activating Death Domain) (Niwa et al., 2008). Kinesin-1 transports a number of different fast axonal transport cargoes including mitochondria and a variety of vesicular and non-vesicular cargoes such as lysosomes, signalling endosomes (e.g. brain-derived neurotrophic factor (BDNF) and tropomyosin receptor kinase (Trk) B (TrkB) vesicles), amyloid precursor protein (APP) vesicles, AMPA vesicles, and mRNA/protein complexes. Kinesin-1 also mediates the slow axonal transport of cytoskeletal cargoes such as microtubules and neurofilaments (reviewed in Hirokawa et al., 2010). KIF1A and KIF1Bβ motors transport synaptic vesicle precursors (Okada et al., 1995), signalling endosomes such as TrkA vesicles (Tanaka et al., 2016), and the autophagy protein ATG9 (Stavoe et al., 2016). KIF1Bα has also been proposed to drive anterograde transport of mitochondria (Nangaku et al., 1994).

In contrast to the multiple kinesins that drive anterograde transport, retrograde transport is almost exclusively mediated by a single cytoplasmic dynein. Cytoplasmic dyneins are members of the ATPases associated with diverse cellular activities (AAA +) family of ATPase proteins. They are sub-divided into cytoplasmic dynein 1 and 2, with cytoplasmic dynein 1 being the main retrograde molecular motor in neurons. Cytoplasmic dynein 1 (hereafter referred to as dynein) is composed of two homodimerised dynein heavy chains (DHCs) and multiple dynein intermediate (DIC), dynein light intermediate (DLIC), and light chains (LC) (reviewed in King, 2012). The assembly of these polypeptides forms a ~ 1.5 MDa protein complex whose functions, cargo binding and localisation are regulated by adapter complexes including dynactin, Bicaudal D2 (BICD2), lissencephaly 1 (LIS1), nuclear distribution protein (NUDE or NDE) and NUDE-like (NUDEL or NDEL). The ~ 1 MDa dynactin complex contains p150Glued which interacts with a short actin-like Arp1 filament and various additional dynactin subunits including p50/dynamitin, p62, CapZ, p27, p25, and p24. p150Glued associates with dynein via the DICs and also directly binds to microtubules; through its cargo-binding domain p150Glued binds a number of vesicular cargo adapters, including sorting nexin 6 (SNX6), huntingtin-associated protein 1 (HAP1) and JIP1 (reviewed in Kardon and Vale, 2009, Fu and Holzbaur, 2014).

## 2 Axonal transport defects in ALS

ALS, the most common form of MND, is an adult onset and progressive neurodegenerative disorder caused by selective injury and death of upper motor neurons in the motor cortex and lower motor neurons in the brain stem and spinal cord. Degeneration of motor neurons leads to progressive muscle wasting followed by paralysis and usually culminates in death through respiratory failure. ALS has an incidence of 2 per 100,000 and a mean age of onset of 55–65 years. The average survival is approximately 3 years from symptom onset (reviewed in Kiernan et al., 2011). An estimated 10% of ALS is inherited, usually in an autosomal dominant fashion (familial ALS), but most ALS cases have no clear genetic basis and occur seemingly random in the population (sporadic ALS). Several genes have been associated with familial ALS, including *superoxide dismutase 1* (*SOD1*) (~ 12% of familial cases), *TAR DNA binding protein* (*TARDBP*; TDP-43) (~ 4%), *Fused in sarcoma* (*FUS*) (~ 4%), and *C9orf72* (~ 40%) (reviewed in Renton et al., 2014). The causes of motor neuron degeneration appear multifactorial. From research mostly on familial ALS cases and animal models a number of possible pathogenic mechanisms underlying motor neuron degeneration have emerged including oxidative stress, mitochondrial dysfunction, misfolded protein toxicity/autophagy defects, RNA toxicity, excitotoxicity, and defective axonal transport (reviewed in Ferraiuolo et al., 2011, De Vos et al., 2008, Millecamps and Julien, 2013).

### 2.1 Axonal pathology

**Fig. 1:**
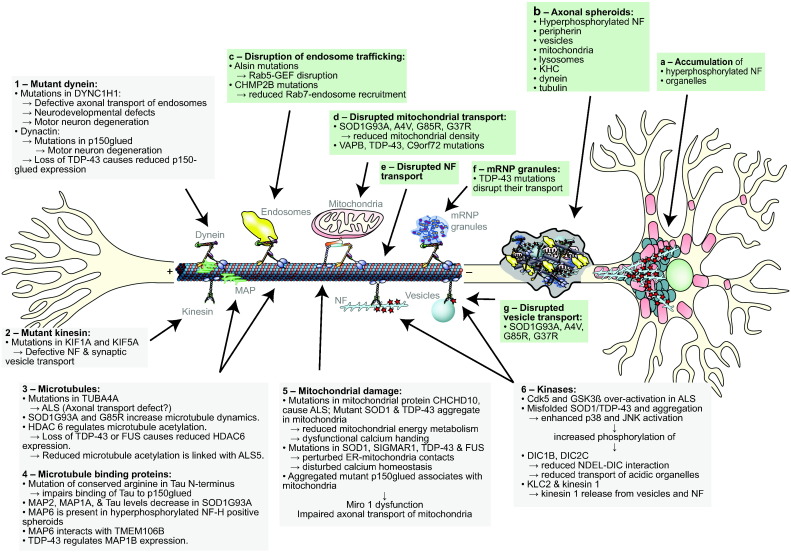
Axonal transport defects in ALS and underlying mechanisms. The axonal transport of various organelles has been shown to be defective in a number of ALS models and in ALS patients (a–g). A number of proposed molecular mechanisms underlying defective transport are indicated (1–6). See text for details.

Early evidence for axonal transport defects in ALS came from electron microscopy and neuropathological studies of post-mortem ALS cases that revealed abnormal accumulations of phosphorylated neurofilaments (a pathological hallmark of ALS), mitochondria and lysosomes in the proximal axon of large motor neurons (Okada et al., 1995, Hirano et al., 1984b, Hirano et al., 1984a, Rouleau et al., 1996) and axonal spheroids containing a variety of vesicles, lysosomes, and mitochondria as well as neurofilaments and microtubules (Sasaki and Iwata, 1996, Corbo and Hays, 1992) (Fig. 1). Consistent with damage to the axonal transport machinery as an underlying cause, hyperphosphorylated neurofilament heavy polypeptide (NF-H) positive spheroids stained strongly for KHC but, interestingly, not dynein (Toyoshima et al., 1998).

(Figure adapted with permission from Annual Review of Neuroscience, Vol. 31, De Vos, K. J., Grierson, A. J., Ackerley, S., and Miller, C. C., Role of axonal transport in neurodegenerative diseases, p151–173, Copyright © 2008 by Annual Reviews.)

Direct evidence for axonal transport defects in ALS was obtained following the development of mutant SOD1 transgenic mouse strains as mammalian animal models of ALS. Sciatic nerve ligation in SOD1G93A transgenic mice revealed a significant reduction of immune-reactive kinesin-1 on the proximal side of the ligation in both younger asymptomatic and older presymptomatic transgenic mice whereas a marked reduction in dynein immunoreactivity was apparent only in the presymptomatic mice (Warita et al., 1999). Both defects correlated with significant spinal motor neuron loss, reduced myelinated fibre densities in the sciatic nerve, and muscle pathology (Warita et al., 1999). Metabolic labelling experiments revealed a significant reduction in the slow anterograde transport of cytoskeletal components months before the onset of neurodegeneration in SOD1G37R transgenic mice (Williamson and Cleveland, 1999) while both slow and fast axonal transport were found to be impaired in SOD1G93A transgenic mice (Zhang et al., 1997).

### 2.2 Endosome trafficking and retrograde signalling

Detailed analysis of axonal transport of specific cargoes in primary neurons and in vivo further confirmed these early studies. Time-lapse recording of a fluorescently labelled fragment of the tetanus toxin TeNT H_C_ which is transported in the same compartment as neurotrophins, revealed defective dynein-mediated retrograde transport in motor neurons isolated from SOD1G93A transgenic embryos (Kieran et al., 2005) and in vivo in the intact sciatic nerve of presymptomatic SOD1G93A transgenic mice (Bilsland et al., 2010). Further evidence for the involvement of perturbed dynein-mediated retrograde axonal transport was provided by examining the transport of a neurotracer to the soma of spinal motor neurons following its injection to the gastrocnemius muscle in SOD1G93A transgenic mice. This investigation demonstrated a significant reduction in retrograde axonal transport, which temporally correlated with disease progression (Ligon et al., 2005). Similarly, direct time-lapse recordings of fluorescently labelled TrkB vesicles revealed defective retrograde transport in SOD1G93A expressing neurons (Bilsland et al., 2010). Interestingly, mutations in alsin (ALS2), which cause juvenile-onset ALS, disturb its Rab5-GEF activity and consequently disrupt Rab5-dependent endosome trafficking and AMPA receptor trafficking (Hadano et al., 2006, Lai et al., 2006, Devon et al., 2006, Lai et al., 2009). Since retrograde neurotrophin trafficking requires Rab5 activity (Deinhardt et al., 2006) alsin mutations may thus cause neurodegeneration by inhibition of retrograde axonal transport. Along the same lines it has been shown that ALS mutant charged multivesicular body protein 2B (CHMP2B) impairs recruitment of Rab7 to endosomes (Urwin et al., 2010). Because Rab7 is also required for retrograde neurotrophin signalling (Deinhardt et al., 2006), disrupted retrograde trafficking may explain the neuronal inclusion formation and axonal degeneration in mutant CHMP2B transgenic mice (Ghazi-Noori et al., 2012). In contrast to the retrograde-specific inhibition of axonal transport of TrkB signalling endosomes, time-lapse recording of EGFP-APP labelled vesicles revealed reduced transport in both anterograde and retrograde directions in mutant SOD1G93A, A4V, G85R or G37R transfected cortical neurons (De Vos et al., 2007). Nevertheless, this data indicates a possible role for disrupted retrograde signalling in ALS.

### 2.3 Mitochondrial trafficking

Live microscopy revealed reduced anterograde but not retrograde axonal transport of fluorescently labelled mitochondria in cultured cortical neurons expressing ALS mutant SOD1G93A, A4V, G85R or G37R and in embryonic motor neurons expressing SOD1G93A (De Vos et al., 2007). This defect was later confirmed in vivo by time-lapse measurements in single axons in the intact sciatic nerve of presymptomatic SOD1G93A transgenic mice (Bilsland et al., 2010, Magrané et al., 2014) and rats (Magrané et al., 2012). In cultured neurons, the transport deficit resulted in depletion of mitochondria from axons and a concomitant increase in inter-mitochondrial distance (De Vos et al., 2007). In vivo in motor neurons of early symptomatic SOD1G37R and SOD1G85R transgenic mice the number of axonal mitochondria was reduced and their distribution was no longer homogeneous throughout the axon (Vande Velde et al., 2011) and in SOD1G93A transgenic mice mitochondria were found in abnormal clusters along the axon (Magrané et al., 2014). Likewise, reduced axonal transport correlated with decreased mitochondrial density in the motor axons of SOD1G93A transgenic rats (Magrané et al., 2012).

One group reported axonal transport defects in wild type SOD1 transgenic mice that show no neurodegeneration, and no axonal transport defects in SOD1G85R transgenic mice (Marinkovic et al., 2012). However, compared to SOD1G93A transgenic mice the onset of the transport defect is later in wild type SOD1 transgenic mice, 2 months after birth in wild type SOD1 transgenic mice compared to postnatal day 20 in SOD1G93A transgenic mice, and only reaches levels comparable to SOD1G93A at 4 months of age (Marinkovic et al., 2012). It has been reported that wild type SOD1 transgenic mice exhibit signs of premature aging (Avraham et al., 1991, Avraham et al., 1988). Thus, it is possible that the late transport defect in wild type SOD1 transgenic mice is linked to the reductions in transport that have been observed in aging mice (Milde et al., 2015). The lack of axonal transport defects in SOD1G85R transgenic mice is in contrast with the reduction in the number of axonal mitochondria and the skewed distribution of mitochondria observed by others (Vande Velde et al., 2011), but may be due to the fact that unlike other mutant SOD1 transgenic mice the SOD1G85R transgenic mice only express low levels of the unstable SOD1G85R protein and the mice tend to remain healthy for most of their lifespan, only succumbing to the disease approximately one week before death (Bruijn et al., 1997).

Defects in mitochondrial transport are not limited to SOD1-related ALS. Overexpression of the ALS mutant vesicle-associated membrane protein-associated protein B (VAPB) VAPBP56S caused a selective block in anterograde transport of mitochondria (Morotz et al., 2012). Similarly, overexpression of wild-type TDP-43 and to a greater extent ALS mutant TDP-43Q331K or M337V reduced mitochondrial transport and mitochondrial density in primary motor neurons (Wang et al., 2013). Disruption of axonal mitochondrial transport was also observed in vivo in ALS mutant TDP-43A315T transgenic mice (Magrané et al., 2014) and wild-type TDP-43 and mutant TDP-43M337V overexpressing mice exhibited mitochondrial aggregation consistent with transport defects (Xu et al., 2011, Xu et al., 2010). It has to be noted that in contrast to these studies, Alami et al. did not find disruption of axonal transport of mitochondria in cortical neurons expressing wild type or mutant TDP-43M337V or A315T at 5–7 days in culture (Alami et al., 2014). Moreover, expression of wild type or TDP-43M337V did not affect mitochondrial transport in *Drosophila* motor axons, although in the same study TDP-43M337V did reduce the KIF1A-dependent motility of dense-core vesicles visualised using NPY-GFP (Baldwin et al., 2016). Possibly, different model systems, neuronal types, and experimental conditions may explain these opposing results.

Expression of either wild type human FUS or the ALS-associated FUS-P525L mutant in *Drosophila* motor neurons reduced both motility and processivity of mitochondrial axonal transport (Chen et al., 2016) but this was not observed by others (Baldwin et al., 2016). Interestingly Baldwin et al. did find that expression the fly homolog of FUS, cabeza (caz) and cazP398L, a pathogenic equivalent of human FUS-P525L, inhibited mitochondrial transport (Baldwin et al., 2016). The same authors explored the effects of transgenic expression of C9orf72 GGGGCC (G4C2) repeat expansion constructs on axonal transport and found that a non-pathogenic repeat length (G4C2-3) had no effect on mitochondrial transport while expression of 36 repeats (G4C2-36) which were previously shown to cause neurotoxicity in this model, caused a decrease in the number of motile mitochondria (Baldwin et al., 2016). The glycine-alanine (GA) dipeptide repeat protein (DPR) produced by repeat-associated non-ATG (RAN) translation of the pathogenic C9orf72 GGGGCC expanded repeats has been shown to interact with Unc119, which is involved in trafficking of myristolated proteins in *Caenorhabditis* (May et al., 2014). It remains to be determined whether sequestration of Unc119 to GA DPRs causes axonal transport defects in mammalian neurons.

### 2.4 mRNP granules

TDP-43 itself is actively transported in motor neuron axons (Fallini et al., 2012). It binds to G-quadruplex-containing mRNAs and assembles into cytoplasmic mRNP granules that undergo bidirectional axonal transport and facilitate delivery of mRNA for local translation (Ishiguro et al., 2016, Alami et al., 2014). Pathogenic mutations in TDP-43 (M337, A315T) caused reductions in net displacements in both anterograde and retrograde directions of TDP-43 granules in transfected mouse cortical neurons and this was caused by reduced motility and increased reversal of direction. In contrast, in vivo examination of *Drosophila* motor axons revealed that TDP-43M337V and TDP-43A315T granules exhibited selectively impaired anterograde movement (Alami et al., 2014). Similarly, in stem cell-derived motor neurons from ALS patients bearing three different ALS-causing mutations in TDP-43 (G298S, A315T, M337V), TDP-43-mediated anterograde transport of *NEFL* mRNA was significantly decreased approximately 10 days after plating and this transport deficit progressively worsened with time in culture (Alami et al., 2014).

Together these data provide strong evidence for a potential involvement of defective axonal transport in disease development and/or progression long before symptom onset. Indeed, axonal transport defects are one of the earliest defects recorded in ALS, suggesting that they may be a key pathogenic event in disease.

## 3 Molecular mechanisms of axonal transport defects in ALS

The underlying cause of axonal transport defects in ALS is not fully understood. A small number of cases involve mutations in the axonal transport machinery; these cases definitively link axonal transport defects to disease. Several mechanisms by which axonal transport may be perturbed in sporadic ALS and familial ALS with mutations in non-axonal transport genes have been proposed mostly based on studies of mutant SOD1-related ALS. These include reductions in microtubule stability, mitochondrial damage, pathogenic signalling that alters phosphorylation of molecular motors to regulate their function or of cargoes such as neurofilaments to disrupt their association with motors, and protein aggregation (Table 1) (Fig. 1).

**Table 1:** Potential impact of MND-associated genes on the axonal transport pathway. Pathogenic variants of the proteins in this table have been linked to disrupted axonal transport.

Gene	Protein	Potential consequence of mutation on axonal transport	Disease
*ALS2*	Alsin	Impaired endocytic trafficking, signalling endosomes	FALS (ALS2)
*C9orf72*	C9orf72	Defective transport of mitochondria	FALS (ALS-FTD1); SALS; FTD
*CHMP2B*	Charged multivesicular body protein 2B	Impaired endocytic trafficking, signalling endosomes	FALS (ALS17); SALS; FTD
*DCTN1*	Dynactin 1 (p150, glued homolog, *Drosophila*)	Altered axonal transport and vesicle trafficking, impaired signalling endosome trafficking	FALS; SALS; HMN7B; PMA; PSP; Perry syndrome
*FUS*	RNA-binding protein FUS	Defective transport of mitochondria, aberrant microtubule acetylation	FALS (ALS6); SALS
*SPG11*	Spatacsin	Axonal destabilisation, reduced tubulin acetylation, reduced anterograde vesicle transport	FALS (ALS5); HSP (SPG11)
*SOD1*	Superoxide dismutase 1	Impaired transport of mitochondria, microtubule stability, modulation of motor proteins via p38 MAP kinase	FALS (ALS1); SALS
*TARDBP*	TAR DNA-binding protein 43	Defective transport of mitochondria and mRNP granules; reduced expression of dynactin 1; aberrant microtubule stability/acetylation,	FALS (ALS10); SALS
*TUBA4A*	Tubulin, alpha 4a	Destabilisation of microtubules, general transport defect?	FALS
*VAPB*	Vesicle-associated membrane protein-associated protein B	Impaired transport of mitochondria and vesicles	FALS (ALS8); SMA; PMA
*KIF1A*	Kinesin Family Member 1A	Reduced kinesin-3 mediated transport	HSP (SPG30)
*KIF5A*	Kinesin heavy chain	Reduced kinesin-1 mediated transport, impaired neurofilament transport	HSP (SPG10)
*SPAST*	Spastin	Destabilisation of microtubules, impaired transport of mitochondria and vesicles	HSP (SPG4)
*AR*	Androgen receptor	Defective retrograde and anterograde transport, modulation of motor proteins via JNK	SBMA

Abbreviations: FALS, familial ALS; SALS, sporadic ALS; SMA, spinal muscular atrophy; SBMA, spinal and bulbar muscular atrophy; PMA, progressive muscular atrophy; FTD, frontotemporal dementia.

### 3.1 Mutations in axonal transport machinery genes as a primary cause of disease

#### 3.1.1 Dynein

Evidence that dysfunctional dynein/dynactin mediated axonal transport is sufficient to cause motor neuron degeneration first came to light when LaMonte et al. showed that disruption of dynein/dynactin interaction by postnatal overexpression of p50/dynamitin, a 50-kDa subunit of dynactin encoded by *DCTN2*, caused reduced axonal transport in motor neurons and consequently led to a late-onset progressive motor neuron disease phenotype in the transgenic mice (LaMonte et al., 2002). This was followed by several studies showing that loss-of-function mutations in *DCTN1*, which encodes the p150Glued subunit of the dynactin complex, cause a slowly progressive autosomal dominant distal hereditary motor neuropathy with vocal paresis (HMN7B) and ALS (Puls et al., 2003, Puls et al., 2005, Munch et al., 2004, Munch et al., 2005). The autosomal dominant G59S mutation that causes HMN7B is in the cytoskeleton-associated protein glycine-rich (CAP-Gly) domain of p150Glued (residues 48-90). This domain is involved in binding to microtubules and end binding protein 1 (EB1). The G59S mutation has been shown to reduce the binding affinity of p150Glued for microtubules, probably as a result of aberrant folding of the CAP-Gly domain and aggregation of mutant p150Glued (Yan et al., 2015, Puls et al., 2003, Levy et al., 2006). Interestingly these p150Glued G59S aggregates associated with mitochondria (Levy et al., 2006). It is not clear what the significance of this association is but it may directly or indirectly affect the axonal transport of mitochondria.

Homozygous p150Glued G59S knock-in embryos are not viable and the heterozygous mice develop late-onset MND-like phenotypes including abnormal gait, spinal motor neuron loss, increased reactive astrogliosis, and accumulation of cytoskeletal and synaptic vesicle proteins at neuromuscular junctions (Lai et al., 2007). A transgenic mouse model of p150Glued G59S exhibited similar phenotypes (Laird et al., 2008). Other disease causing autosomal dominant mutations in the CAP-Gly domain of p150Glued involve substitution of phenylalanine 52, lysine 56, glycine 71, threonine 72 or glutamine 74. Similar to the G59S mutation, the F52L and K56R mutations reduce the microtubule binding affinity of p150Glued but cause late-onset parkinsonism and frontotemporal atrophy or progressive supranuclear palsy (PSP) (Araki et al., 2014, Gustavsson et al., 2016) while residues 71, 72, and 74 which are within or close to the GKNDG microtubule binding motif of the CAP-Gly domain (residues 68–72) also reduce the binding affinity of p150Glued for microtubules but cause Perry syndrome with neuronal inclusions containing TDP-43 (Farrer et al., 2009). Other p150Glued variants including T1249I, M571T, R785W, and R1101K have been reported as possible risk factors for ALS, but further research is required to establish their role in disease (Munch et al., 2004, Munch et al., 2005, Vilariño-Güell et al., 2009). It is nonetheless clear that mutations in the *DCTN1* gene cause a group of neurological disorders with overlapping clinical and/or neuronal cell pathologies.

Coinciding with the discovery that p150Glued G59S causes HMN7B (Puls et al., 2003), Hafezparast et al. demonstrated that single point mutations in the *Dync1h1* gene, which encodes DHC, cause autosomal dominant motor function defects and motor neuron degeneration in the Legs at odd angles (*Loa*) and Cramping 1 (*Cra1*) mouse strains (Hafezparast et al., 2003). The *Loa* and *Cra1* mutations lead to F580Y and Y1055C amino acid substitutions in DHC, respectively. Heterozygous *Loa* and *Cra1* mice display a limb grasping/clenching phenotype and a progressive movement deficit characterised by a low-based reptilian-like gait. The severity of this abnormal way of walking increases as the animals age (Hafezparast et al., 2003). Heterozygous *Loa* mice also show a severe loss of proprioceptive sensory neurons (Chen et al., 2007, Ilieva et al., 2008). Homozygous *Cra1* and *Loa* mice are not viable with *Cra1* homozygosity being embryonic lethal and *Loa*/*Loa* mice die within a day after birth as a result of the loss of > 90% of their spinal cord motor neurons by E18. Cultured motor neurons isolated from Loa embryos exhibit significantly reduced retrograde axonal transport and aberrant extracellular signal-regulated kinases (ERK) 1/2 and c-Fos expression (Garrett et al., 2014, Hafezparast et al., 2003).

The F580Y mutation in DHC increases its affinity for DICs and DLICs while reducing association of dynactin to dynein (Deng et al., 2010). Thus, impaired transport of cargoes such as signalling endosomes which attach to dynein via dynactin may be explained by reduced dynactin-dynein interaction while reduced motor function is predicted to disturb retrograde transport more generally (Ori-McKenney et al., 2010). Interestingly, the human variantsM581L and I584L that cause a childhood form of motor neuron disease known as spinal muscular atrophy, lower extremity-predominant 1 (SMALED1), are only 1 and 2 amino acids, respectively, from the F580Y substitution in the *Loa* mouse strain (Scoto et al., 2015; reviewed in Schiavo et al. (2013)). It is not clear why these and several other mutations within different domains of DYNC1H1 do not appear to play a more conspicuous role in the pathogenesis of ALS. One explanation could be that these mutations are pathologically detrimental to mainly long motor neurons and therefore spare other motor neuronal pools, degeneration of which tips the balance toward development of ALS.

Finally, dysregulation of transcription in a mouse model of the MND spinal and bulbar muscular atrophy (SBMA) harbouring a pathogenic expanded trinucleotide CAG repeat in the androgen receptor (AR) protein leads to reduced levels of p150Glued mRNA, which is accompanied by impaired retrograde axonal transport (Katsuno et al., 2006). Moreover, loss of TDP-43 led to decreased expression of p150Glued and impaired autophagosome-lysosome fusion, which could be rescued by transfecting the cells with p150Glued (Xia et al., 2015a, Xia et al., 2015b). Thus, in some cases dynein function appears to be directly affected by disease-associated downregulation of p150Glued expression.

#### 3.1.2 Kinesin

As is the case for dynein, disruption of kinesin can cause neurodegeneration. Conditional knockout of *Kif5a* in mice caused paralysis and neurodegeneration concomitant with a reduction in neurofilament axonal transport (Xia et al., 2003). Similarly, disruption of *Kif1a* in mice led to severe motor and sensory defects and lethality within one day of birth. *Kif1a* knockout reduces transport of synaptic vesicle precursors and as a consequence causes decreases in synaptic vesicle density accompanied by neuronal degeneration in vivo and in cultured neurons (Yonekawa et al., 1998).

Mutations in kinesin-1 or KIF1A have not directly been linked to ALS, but mutations in *KIF5A and KIF1A* have been identified in hereditary spastic paraplegia (HSP) forms of MND (Fichera et al., 2004, López et al., 2015, Muglia et al., 2014, Citterio et al., 2015, Erlich et al., 2011, Lee et al., 2015a). Both KIF5A and KIF1A mutations are located in the motor or neck domains and appear to be loss-of-function variants (Ebbing et al., 2008, Citterio et al., 2015, Erlich et al., 2011, Lee et al., 2015a).

#### 3.1.3 α-Tubulin

Microtubules play a pivotal role in the development and maintenance of neuronal cell structures and functions and they are the essential tracks for both fast and slow long-distance axonal transport. As such, it is not surprising that perturbations in the integrity of the microtubule cytoskeleton have been linked with several neurodegenerative diseases including MND and this is exemplified by the disease-causing mutations in α-tubulin and associated proteins (reviewed in El-Kadi et al., 2007, Clark et al., 2016).

Several variants of the α-tubulin gene *TUBA4A* that destabilise the microtubule network and diminish its re-polymerisation capability have been identified as a possible cause of ALS (Smith et al., 2014). Whether these mutations affect axonal transport has not yet been determined, but since axonal transport prefers stable microtubules (Cai et al., 2009, Reed et al., 2006) it is likely that they will have a detrimental effect. In this context, it is noteworthy that a missense mutation in the *tubulin-specific chaperone E* (*Tbce*) gene that causes motor neuron degeneration in the *progressive motor neuronopathy (pmn)* mouse strain, a model of human MND, causes microtubule loss similar to that induced by human ALS-linked *TUBA4A* mutations, and axonal transport defects (Bommel et al., 2002, Martin et al., 2002, Schäfer et al., 2016). Finally, mutations in spastin, a microtubule severing protein, which are the most common cause of HSP (Hazan et al., 1999) impair microtubule dynamics (Wood et al., 2006, Trotta et al., 2004, McDermott et al., 2003, Evans et al., 2005, Errico et al., 2002) and axonal transport of mitochondria and APP vesicles (Kasher et al., 2009, Tarrade et al., 2006).

### 3.2 Pathogenic signalling as a cause of axonal transport defects

Although mutations in the molecular machinery of axonal transport unequivocally link transport defects to neurodegeneration and disease, these mutations are very rare. Nevertheless, as summarised above, axonal transport defects are a common occurrence across several models of familial ALS and have been described in sporadic ALS. In this section we summarise the possible mechanisms underlying axonal transport defects in these cases.

#### 3.2.1 Microtubule stability

Microtubules are dynamic structures that may undergo assembly or disassembly by a mechanism called dynamic instability (reviewed in Matamoros and Baas, 2016). Some microtubules are more stable than others resulting in two populations referred to as labile and stable microtubules. The stability of microtubules is mainly regulated by microtubule associated proteins (MAPs) that bind along the length of the microtubule, or by capture of the plus ends by for instance protein complexes in the cell cortex. Several MAPs have been shown to stabilise microtubules in neurons, including tau, MAP2 and MAP1B. Tau, which is mainly expressed in neurons where it localises to axons, is of particular interest in the context of neurodegeneration and axonal transport. Mutations in tau have been shown to cause frontotemporal dementia with parkinsonism linked to tau mutations on chromosome 17 (FTDP-17T) and neurofibrillary tangles which mainly consist of hyperphosphorylated tau are a pathological hallmark of Alzheimer's disease (Grundke-Iqbal et al., 1986; reviewed in Iqbal et al., 2016). Tau has been shown to regulate the axonal transport of several cargoes, including mitochondria, possibly by regulating motor/microtubule interactions and/or by stabilising microtubules (Ebneth, 1998, Stamer et al., 2002, Seitz, 2002, Trinczek et al., 1999). In a further link between neurodegeneration and defective axonal transport FTD-mutant tau inhibits axonal transport (Zhang et al., 2004, Rodríguez-Martín et al., 2016, Gilley et al., 2012). In addition, the PSP-associated R5L and R5H mutants in the N-terminal projection domain of tau disrupt its interaction with the C-terminus of p150Glued (Magnani et al., 2007). MAP1B has been implicated in the retrograde transport of mitochondria (Jimenez-Mateos et al., 2006) and disruption of FUTSCH/MAP1B in *Drosophila* caused mitochondrial transport defects and progressive neurodegeneration (Bettencourt da Cruz et al., 2005). Interestingly, MAP1B mRNA is a translational target of TDP-43 and restoring its expression is protective in a *Drosophila* model of TDP-43-related ALS (Coyne et al., 2014, Godena et al., 2011).

In addition to the labile and stable microtubule populations, a population of ultra-stable, virtually non-dynamic, so-called cold-stable microtubules have been identified. Cold-stable microtubules are enriched in axons and are made up by tubulin that has been post-translationally polyaminated (Song et al., 2013a). Additional tubulin modifications that have been linked to microtubule stability are α-tubulin acetylation and detyrosination, but it appears that these modifications accumulate on longer-lived more stable microtubules rather than stabilise microtubules per se. Hence the presence of acetylated or detyrosinated α-tubulin is a marker for stable microtubules. Post-translational tubulin modifications have been linked to regulation of kinesin-1 mediated axonal transport. Thus, kinesin-1 appears to preferentially bind to acetylated and/or detyrosinated microtubules. Microtubule acetylation has been shown to stimulate kinesin-1-mediated transport (Hammond et al., 2010, Reed et al., 2006), while tubulin detyrosination appears to direct kinesin-1 to the axon (Konishi and Setou, 2009). If and how post-translational modifications of tubulin affect dynein-mediated transport is less clear, but increasing α-tubulin acetylation has been shown to cause recruitment of dynein to microtubules (Dompierre et al., 2007) and in case of axonemal dynein, microtubule acetylation increased motility (Alper et al., 2014). Microtubule acetylation occurs primarily on the epsilon amino group of the lysine at position 40 (K40) of α-tubulin by α-tubulin acetyl transferase 1 (αTAT1, also known as MEC17) (Shida et al., 2010, Akella et al., 2010), and deacetylation is mediated by histone deacetylase 6 (HDAC6) (Hubbert et al., 2002) and Sirtuin-2 (SIRT2) (North et al., 2003). Interestingly loss of TDP-43 or FUS has been shown to reduce HDAC6 expression (Kim et al., 2010), suggesting that ALS-associated TDP-43 and FUS dysfunction may affect axonal transport via changes to microtubule acetylation.

Measurement of in vivo microtubule polymerisation/depolymerisation rates using mass spectrometry analysis of ^2^H_2_O-labelled tubulin revealed an increase in microtubule dynamics in presymptomatic SOD1G93A transgenic mice, which correlated with impaired slow axonal transport and progressively increased with disease. In addition, hyperdynamic microtubule subpopulations were found in the lumbar segment of the spinal cord (where motor neuron pathology occurs) and cerebral cortex, and in the peripheral motor and sciatic mixed nerves, but not in sensory nerves (Fanara et al., 2007). Direct identification of dynamic microtubules by live imaging of EB3-YFP also identified increased microtubule dynamics in intercostal axons of *Thy1*:EB3-YFP–SOD1G93A and G85R transgenic mice (Kleele et al., 2014).

Mutant SOD1A4V, G85R and G93A but not wild type SOD1 have been shown to interact with tubulin and to affect microtubule stability in vitro (Kabuta et al., 2009), providing a possible explanation for decreased microtubule stability in vivo. Alternatively, mutant SOD1-associated reductions in microtubule stability may involve excitotoxicity-related increases in intracellular calcium levels (reviewed in Grosskreutz et al., 2010) that induce depolymerisation of microtubules (Furukawa and Mattson, 1995), or oxidative stress (reviewed in Bozzo et al., 2016), which has been shown to affect microtubule stability, albeit in non-neuronal cells (Kratzer et al., 2012, Drum et al., 2016). Further insults may involve changes to MAPs. MAP2, MAP1A, and tau levels are reported to be reduced in the spinal cord of pre-symptomatic SOD1G37R transgenic mice (Farah et al., 2003), and tau hyperphosphorylation, which is predicted to reduce tau binding to microtubules and hence lower microtubule stability (Wagner et al., 1996) was reported in the same mouse model (Nguyen et al., 2001).

Indications that microtubule stability may also be affected in sporadic ALS come from the observation that in post-mortem spinal cord and brain tissue sections of sporadic ALS cases hyperphosphorylated NF-H positive spheroids also show positive staining for microtubule associated protein 6 (MAP6) (Letournel et al., 2003). MAP6, which is also known as stable tubule only polypeptide (STOP), protects microtubules from cold-induced depolymerisation (Delphin et al., 2012) and is preferentially associated with stable microtubules in neurons (Slaughter and Black, 2003). Its abnormal accumulation in the spheroids suggest disruption of stable microtubules and consequently disrupted transport, which may be a contributory factor in sporadic ALS. Interestingly MAP6 also interacts with TMEM106B, a major risk factor of frontotemporal dementia (FTD) and a modifier of C9orf72-associated ALS and FTD that is involved in axonal transport of lysosomes (Schwenk et al., 2014, van Blitterswijk et al., 2014, Gallagher et al., 2014, Van Deerlin et al., 2010). Finally, a reduction in the levels of acetylated tubulin has been linked to axonal instability and axonal transport defects in familial ALS (ALS5) and HSP (SPG11) caused by mutations is spatacsin (Perez-Branguli et al., 2014).

#### 3.2.2 Mitochondrial damage

Mitochondria play a pivotal role in many cellular events including energy metabolism and calcium handling. The latter is of special importance for motor neurons that rely greatly on mitochondria for calcium buffering (reviewed in Grosskreutz et al., 2010). Furthermore, calcium handling and ATP production by mitochondria are intimately linked because mitochondrial calcium activates the rate-limiting enzymes of the Krebs cycle and thereby increases oxidative phosphorylation and ATP synthesis to match local energy demand. Evidence suggests that both reduced mitochondrial energy metabolism and dysfunctional calcium handling are likely to be main contributors to the axonal transport defects observed in ALS. Moreover, it is likely that impaired transport of mitochondria themselves and concomitant depletion of mitochondria from axons (De Vos et al., 2007, Vande Velde et al., 2011, Magrané et al., 2012, Wang et al., 2013) further exacerbates any defects.

Damage to mitochondria is a well-documented, early phenomenon in ALS (reviewed in Carrì et al., 2016, Grosskreutz et al., 2010, Tan et al., 2014). In SOD1 or TDP-43-associated familial ALS mitochondrial damage appears to be directly linked to pathological accumulations of aggregated ALS mutant SOD1 or TDP-43 in mitochondria (Magrané et al., 2009, Igoudjil et al., 2011, Pickles et al., 2013, Pickles et al., 2016, Israelson et al., 2010, Li et al., 2010, Pasinelli et al., 2004, Liu et al., 2004, Cozzolino et al., 2009, Wang et al., 2016). Whether aggregated TDP-43 also accumulates in mitochondria in sporadic ALS is not yet clear. ALS mutant SOD1 has been shown to specifically interact with spinal cord mitochondria via direct interaction with voltage-dependent anion channel 1 (VDAC1) and this accumulation is sufficient and necessary to damage mitochondria (Israelson et al., 2010). Accumulation of ALS mutant TDP-43 in mitochondria appears to be mediated by internal mitochondrial targeting sequences in TDP-43 (Wang et al., 2016). Mutant SOD1 and TDP-43-mediated damage to mitochondria is believed to severely impair the mitochondrial electron transfer chain and ATP synthesis (Mattiazzi et al., 2002, Wang et al., 2016). Overexpression of FUS has been shown to reduce mitochondrial ATP production, but whether ALS mutant FUS accumulates in mitochondria is not clear (Stoica et al., 2016). Mutations in the mitochondrial protein CHCHD10 have been shown to cause familial ALS (Bannwarth et al., 2014). CHCHD10 is localised to contact sites between the inner and outer mitochondrial membrane and mutations disrupt mitochondrial cristae and impair mitochondrial genome maintenance (Genin et al., 2016). It is not clear if CHCHD10 mutants directly affect mitochondrial function, but since assembly and maintenance of the mitochondrial electron transport chain relies on intact cristae (Vogel et al., 2006) and mitochondrial encoded subunits, it is likely that they do. Indeed, disruption of cristae by mitofilin depletion disrupts mitochondrial function (John et al., 2005) and reduces mitochondrial membrane potential and ATP levels (Ding et al., 2015). In agreement, respiratory chain complex I, III and IV deficiency was identified in fibroblasts of a CHCHD10 patient (Bannwarth et al., 2014).

In addition to decreased ATP production, damage to mitochondria has been associated with the disrupted calcium homeostasis observed in in vitro and in vivo models of mutant SOD1, VAPB, TDP-43, and FUS-related ALS (Carrì et al., 1997, Siklós et al., 1998, Morotz et al., 2012, Stoica et al., 2014, Stoica et al., 2016). Compelling evidence suggests that disrupted calcium homeostasis is caused by dysfunctional communication between the endoplasmic reticulum (ER) and mitochondria at mitochondria-associated ER membranes (MAM). Reduced ER/mitochondria contact sites have been observed in mutant SOD1, SIGMAR1, TDP-43, and FUS-related ALS (Stoica et al., 2014, Stoica et al., 2016, Watanabe et al., 2016, Lautenschlager et al., 2013). In contrast overexpression of ALS mutant VAPBP56S increased ER/mitochondria contacts (De Vos et al., 2012), but since in ALS8 patient-derived iPSC neurons VAPB expression is down-regulated because of reduced expression of the VAPBP56S mutant (Mitne-Neto et al., 2011), it is likely that in VAPBP65S-related ALS ER/mitochondria contacts are actually decreased as well. ER interacts with mitochondria via tethering proteins (reviewed in Paillusson et al., 2016), such as the ER protein VAPB that binds to the mitochondrial outer membrane protein PTPIP51 (De Vos et al., 2012). In case of mutant TDP-43 and FUS the reduction in ER/mitochondria contact was the direct result of decreased binding of VAPB to PTPIP51 (Stoica et al., 2014, Stoica et al., 2016). If this is also the case in mutant SOD1 and SIGMAR1-related ALS remains to be determined. Interestingly, the levels of VAPB are reduced in the spinal cord of sporadic ALS cases (Anagnostou et al., 2010), suggesting that disrupted ER-mitochondria communication could be a general feature in ALS and that restoring ER/mitochondria contact may be of therapeutic benefit. In agreement with this, neuronal overexpression of wild-type human VAPB has been shown to slow disease and increase survival in SOD1G93A transgenic mice (Kim et al., 2016).

The outer mitochondrial membrane protein Miro1 has emerged as the main regulator of axonal transport of mitochondria although the remaining transport of mitochondria in Miro1 knockout neurons suggests that at least some mitochondrial transport is Miro1 independent (Stowers et al., 2002, Glater et al., 2006, Russo et al., 2009, Babic et al., 2015, López-Doménech et al., 2016). Possibly Miro2 can partly compensate for the loss of Miro1. Kinesin-1 connects to mitochondria through interaction with Miro1 via the adaptor proteins TRAK1 and 2 (Glater et al., 2006, Brickley et al., 2005, MacAskill et al., 2009a, Brickley and Stephenson, 2011), and dynein has been shown to interact with both Miro1 (Morlino et al., 2014) and TRAK1/2 (van Spronsen et al., 2013). The Miro1/TRAK1 complex further associates with disrupted in schizophrenia 1 (DISC1) and this has been linked to regulation of anterograde mitochondrial transport (Atkin et al., 2011, Ogawa et al., 2014, Norkett et al., 2016), possibly via interaction with the anchoring protein syntaphilin (Park et al., 2016) or NDE1 and glycogen synthase kinase 3β (GSK3β) (Ogawa et al., 2016) or by regulating mitochondrial bioenergetics via interaction with mitofilin and the mitochondrial contact site and cristae organising system (MICOS) complex (Park et al., 2010, Piñero-Martos et al., 2016).

Miro1 is an atypical Rho GTPase comprised of two GTPase domains separated by two calcium-binding *E*-helix-loop-F-helix (EF)-hand motifs, and is anchored in the mitochondrial outer membrane by a C-terminal transmembrane domain (Fransson et al., 2006). Miro1 plays a central role in the regulation of mitochondrial axonal transport in response to calcium and mitochondrial damage (Russo et al., 2009, Babic et al., 2015, Saotome et al., 2008, Weihofen et al., 2009, Wang et al., 2011).

Binding of calcium to the Miro EF-hand motifs halts anterograde transport of mitochondria by regulating the interaction of kinesin-1 with Miro1 such that either kinesin-1 binding to microtubules or to Miro1 is disrupted and this appears an important mechanism to regulate mitochondrial transport in response to physiological stimuli (MacAskill et al., 2009b, Wang and Schwarz, 2009, Stephen et al., 2015). Increased cytosolic calcium levels have been reported in cellular models and in motor neurons from transgenic ALS models (Morotz et al., 2012, Siklós et al., 1998) and have been shown to disrupt transport of mitochondria via Miro1 in VAPBP56S-expressing neurons (Morotz et al., 2012).

In mitophagy, loss of mitochondrial membrane potential or accumulation of misfolded proteins in mitochondria, leads to the stabilisation and activation of the Ser/Thr kinase PINK1 on the outer mitochondrial membrane. PINK1 subsequently phosphorylates ubiquitin on Ser65 which drives recruitment of the cytosolic E3 ubiquitin ligase Parkin to damaged mitochondria. PINK1 further phosphorylates Parkin leading to its full activation. PINK1 also forms a complex with Miro1 and TRAK and phosphorylates Miro1 in response to mitochondrial damage (Weihofen et al., 2009, Wang et al., 2011, Shlevkov et al., 2016). Phosphorylated Miro is targeted for proteasomal degradation in a Parkin-dependent manner and as a result kinesin-1 detaches from the mitochondrial surface and mitochondrial movement is arrested (Wang et al., 2011). In addition, Parkin ubiquitinates other outer mitochondrial membrane substrates, such as mitofusin, to isolate the damaged mitochondria and to recruit autophagy receptors such as NDP52, optineurin (both substrates of TANK-binding kinase (TBK1)) and p62 to deliver the damaged mitochondria to autophagosomes. Interestingly loss-of-function mutations in optineurin, p62 and TBK1 have been shown to cause ALS, and the C9orf72 protein regulates autophagy and interacts with SMCR8 which is itself a TBK1 substrate (Webster et al., 2016a, Webster et al., 2016b, Ugolino et al., 2016, Yang et al., 2016). In agreement with ALS-associated mitochondrial damage leading to PINK1/Parkin-mediated halting of mitochondrial transport, decreased levels of Miro1 have been reported in SOD1G93A and TDP-43M337V transgenic mice as well as in the spinal cord of ALS patients (Zhang et al., 2015) while down-regulation of either PINK1 or Parkin partially rescued the locomotive defects and enhanced the survival rate in transgenic flies expressing FUS (Chen et al., 2016). Interestingly mitochondria remained homogeneously distributed throughout the axons of Miro1 knockout neurons despite a 65% decrease in trafficking (López-Doménech et al., 2016). This is reminiscent of the situation in SOD1G93A expressing neurons where reduced anterograde transport and the resulting loss of axonal mitochondria did not translate into changes in the overall distribution of mitochondria in the axon, but rather caused a compensatory increase in inter-mitochondrial distance (De Vos et al., 2007).

Whether mitochondrial axonal transport defects are part of all ALS is not yet clear, but mitochondrial damage (Sasaki and Iwata, 2007, Allen et al., 2015), dysfunctional calcium metabolism (Curti et al., 1996, Siklos et al., 1996) and reduced expression of Miro1 (Zhang et al., 2015) have been found in sporadic ALS cases. Furthermore, energy defects and reduced calcium buffering capacity caused by reduced numbers of mitochondria in the distal axon, exacerbated by mitochondrial and MAM dysfunction, may explain the selective vulnerability of motor neurons because they are particularly reliant on mitochondria for calcium buffering as a consequence of their relative lack of cytosolic calcium binding proteins (Grosskreutz et al., 2010). One obvious way in which mitochondrial damage and concomitant mitochondrial transport defects and depletion of mitochondria from axons (De Vos et al., 2007, Vande Velde et al., 2011, Magrané et al., 2012, Wang et al., 2013) could affect the transport of other cargoes such as APP vesicles or signalling endosomes is by starving molecular motors of ATP. However, since it has been shown that neuronal BDNF, APP, and TrkB vesicles harbour most glycolytic enzymes and “self-propel” using their own source of glycolytic ATP independent of mitochondria (Hinckelmann et al., 2016, Zala et al., 2013) reduced axonal mitochondrial ATP production may not be sufficient to halt axonal transport. Alternatively, mitochondrial damage and/or lack of axonal mitochondria may affect transport by disturbance of calcium signalling. Indeed, MAP6’s microtubule stabilisation activity is regulated by calcium/calmodulin. Increased calcium is associated with increased MAP6/calmodulin interaction and reduced microtubule binding (Job et al., 1981, Lefèvre et al., 2013). Hence increases in cytosolic calcium caused by mitochondrial dysfunction could destabilise microtubules and consequently impair axonal transport.

#### 3.2.3 Kinase signalling

Axonal transport is regulated by phosphorylation (reviewed in Gibbs et al., 2015). Direct phosphorylation of molecular motors has been shown to affect motor activity and phosphorylation of adapter proteins and cargoes has been shown to affect attachment of motors to cargo. Furthermore, phosphorylation of MAPs has been shown to regulate microtubule stability and hence axonal transport. Several of the kinases involved in the regulation of axonal transport have been associated with ALS.

##### 3.2.3.1 p38 MAP kinase

A number of groups have shown that p38 mitogen-activated protein (MAP) kinase is overactivated in the spinal cord of SOD1G93A transgenic mice and in familial and sporadic human ALS cases (Morfini et al., 2013, Tortarolo et al., 2003, Ackerley et al., 2004, Bendotti et al., 2004, Dewil et al., 2007). Although the precise role of p38 MAP kinase in disease is not fully understood, inhibition of p38 MAP kinase protected mutant SOD1 expressing motor neurons in vitro and in vivo in SOD1G93A transgenic mice, suggesting an active role in the neuropathology of disease (Dewil et al., 2007). The activation of p38 MAP kinase probably involves excitotoxic glutamate signalling (Stevenson et al., 2009, Kawasaki et al., 1997, Jeon et al., 2000, Chen et al., 2003) and protein stress by for example misfolded SOD1 (Bosco et al., 2010).

p38 MAP kinase has been shown to phosphorylate kinesin-1 on serines 175 and 176 and this inhibited translocation of kinesin-1 along axonal microtubules (Morfini et al., 2013) while p38 MAP kinase phosphorylation of KLC inhibited anterograde transport of mitochondria (De Vos et al., 2000). p38 MAP kinase also phosphorylates neurofilament medium polypeptide (NF-M)/NF-H sidearms (Ackerley et al., 2004, Guidato et al., 1996) which slows their transport, probably by reducing neurofilament binding to molecular motors (Ackerley et al., 2003, Jung et al., 2005). Supporting a role for p38 MAP kinase in neurofilament pathology, increased co-localisation of p38 MAP kinase and phosphorylated neurofilaments was observed in degenerating neurons at the onset of disease in SOD1G93A transgenic mice (Bendotti et al., 2004). Interestingly, the anti-glutamatergic drug riluzole, currently the only approved drug for the treatment of ALS, has been shown to prevent p38 MAP kinase activation by excitotoxic glutamate and restore axonal transport of neurofilaments (Stevenson et al., 2009).

Using a monoclonal antibody to misfolded SOD1 (C4F6), Bosco et al. (2010) revealed the presence of a misfolded wild-type SOD1 in post-mortem human spinal cord tissues of 4 out of 9 sporadic ALS cases. Misfolded wild-type SOD1 purified from sporadic ALS tissues inhibited anterograde axonal transport in isolated squid axoplasm assays to the same extend as a familial ALS-associated SOD1H46R mutant, and this was found to involve the activation of p38 MAP kinase and subsequent kinesin-1 phosphorylation (Bosco et al., 2010). A later study using YFP-tagged SOD1G85R revealed that Hsc70 and its nucleotide exchange factor Hsp110 prevented SOD1G85R-induced activation of p38 MAP kinase and the transport defect exerted by mutant SOD1G85R, possibly by enhancing disaggregation of SOD1 (Song et al., 2013b). Interestingly, overexpression of Hsp110 has been shown to markedly increase the life span of YFP-SOD1G85R and SOD1G93A transgenic mice (Nagy et al., 2016).

##### 3.2.3.2 JNK

JNK/c-Jun signalling has been implicated in TDP-43 induced protein toxicity (Suzuki and Matsuoka, 2013, Meyerowitz et al., 2011, Lee et al., 2016, Zhan et al., 2015), and increased amounts of phosphorylated c-Jun have been reported in SOD1G93A transgenic mice (Jaarsma et al., 1996). The latter appears to correlate with an increase in retrograde JNK signalling rather than overall increased activation of JNK in motor neurons (Perlson et al., 2009, Holasek et al., 2005). JNK has also been shown to be activated by glutamate excitotoxicity (Chen et al., 2003, Schwarzschild et al., 1997) but if this is the case in ALS is not clear. Indeed, Dewil et al. (2007), did not find JNK activation in motor neurons and microglia from SOD1G93A transgenic mice (Dewil et al., 2007).

JNK has been shown to modulate both kinesin/microtubule (Morfini et al., 2006, Stagi et al., 2006) and kinesin/cargo interactions (Horiuchi et al., 2007). The former has been linked to JNK-mediated phosphorylation of the kinesin-1 motor domain (Morfini et al., 2006) whereas the latter involves disruption of the binding of the cargo adapter JIP1 to kinesin-1 (Horiuchi et al., 2007). JNK also interacts with dynein via binding of JIP3 to p150Glued and DLIC and this is required for retrograde transport of activated JNK (Drerup and Nechiporuk, 2013, Cavalli et al., 2005, Huang et al., 2011). Whether activated JNK regulates its own retrograde transport is not yet clear. Activated JNK may also have a general effect on axonal transport by regulation of Dishevelled-mediated microtubule stability (Ciani and Salinas, 2007). Changes to the WNT signalling pathway have been described in ALS but if these affect microtubules remains to be determined (Chen et al., 2012b, Yu et al., 2013, Chen et al., 2012a). As was the case for p38 MAP kinase, JNK activation has been linked to misfolded protein stress. Neuropathogenic forms of huntingtin and AR were shown to inhibit axonal transport (Szebenyi et al., 2003) and subsequent studies showed that this inhibition is JNK mediated (Morfini et al., 2009, Morfini et al., 2006). Finally, JNK also phosphorylates NF-M/NF-H sidearms (Ackerley et al., 2000).

Overactivation of GSK3β has been reported in the brain and spinal cord of SOD1G93A transgenic mice and spinal cord samples from sporadic ALS patients (Hu et al., 2003a, Hu et al., 2003b, Yang et al., 2008, Koh et al., 2005). Nevertheless, the involvement of GSK3β in ALS remains controversial. Inhibition of GSK3β was protective in SOD1G93A transgenic mice in some studies (Koh et al., 2007, Feng et al., 2008) but not in others (Gill et al., 2009, Pizzasegola et al., 2009) Moreover, lithium, a known inhibitor of GSK3β, did not show any evidence of benefit on survival in patients with ALS (UKMND-LiCALS et al., 2013).

##### 3.2.3.3 GSK3β

GSK3β negatively regulates axonal transport in a number of ways. GSK3β-mediated phosphorylation of KLC2 has been shown to release kinesin-1 from vesicles in a regulatory pathway that involves Cyclin-dependent kinase 5 (Cdk5) (see below), lemur tyrosine kinase 2 (LMTK2) and protein phosphatase 1 (PP1) (Manser et al., 2012, Morfini et al., 2004, Morfini et al., 2002). Phosphorylation of DIC1B and DIC2C by GSK3β inhibited retrograde transport of acidic organelles by reducing the binding of NDEL1 to DICs (Gao et al., 2015). Furthermore, GSK-3β-dependent phosphorylation of the motor adapter BICD1 is required for its binding to dynein (Fumoto et al., 2006). Since NDEL1, LIS1 and BICD are involved in regulation of dynein function, GSK3β may be affecting multiple retrograde cargoes and this may explain the defects in retrograde transport reported in ALS. NDE1, LIS1 and GSK3β have also been shown to interact with TRAK1 and this interaction is involved in regulating axonal transport of mitochondria. Overexpression of NDE1 increased retrograde transport of mitochondria, while activation of GSK3β stimulated anterograde transport (Ogawa et al., 2016). Consistent with NDE1/LIS1 regulation of mitochondrial transport it was shown that reducing the levels of LIS1 increases mitochondrial trafficking in adult *Drosophila* neurons (Vagnoni et al., 2016). Interestingly mutations in LIS1, NDE1 and BICD2 have all been associated with neurodegeneration (Lipka et al., 2013).

GSK3β is a major tau kinase involved in neurodegeneration (reviewed in Hanger and Noble, 2011, Llorens-Martín et al., 2014). Phosphorylation of tau by GSK3β releases tau from microtubules and destabilises microtubules (Wagner et al., 1996) which can disrupt axonal transport. ALS-associated defects in ER/mitochondria communication are linked to activation of GSK3β (Stoica et al., 2016, Stoica et al., 2014). Thus, GSK3β may also indirectly regulate axonal transport by affecting ER/mitochondria communication as described above. GSK3β has also been described as a neurofilament kinase that affects anterograde neurofilament transport by regulating neurofilament bundling (Lee et al., 2014).

##### 3.2.3.4 Cdk5

Cdk5 is a member of the cyclin-dependent kinase family expressed in post-mitotic cells including neurons. Under normal circumstances Cdk5 is activated by p35, which in turn is phosphorylated by Cdk5 leading to its degradation by the proteasome and subsequent inactivation of Cdk5. Under stress conditions p35 is cleaved by calpain to generate a p25 fragment which retains its Cdk5 activation activity, but lacks the regulatory phosphorylation site, leading to sustained activation of Cdk5 and this has been linked to neurodegeneration (Patrick et al., 1999, Kusakawa et al., 2000, Lee et al., 2000). Transgenic overexpression of p25 in neurons caused MND reminiscent of ALS (Bian et al., 2002) and aberrant activation of Cdk5 has been reported in the spinal cord of mouse models of ALS (Nguyen et al., 2001, Klinman and Holzbaur, 2015, Rao et al., 2016). Consistent with a possible role of p25 dependent overactivation of Cdk5 in ALS, overexpression of the endogenous calpain inhibitor calpastatin delayed disease onset and increased survival of SOD1G93A transgenic mice (Rao et al., 2016). However, since genetic ablation of the Cdk5 activator p35 did not affect the onset and progression of motor neuron disease in SOD1G93A transgenic mice (Takahashi and Kulkarni, 2004), the protective effect of calpastatin may actually derive from its inhibition of MAP2 and neurofilament proteolysis. Cdk5 has been shown to phosphorylate neurofilaments and this regulates their transport (Shea et al., 2004a, Shea et al., 2004b, Ackerley et al., 2003). Cdk5 also regulates anterograde trafficking of vesicles by activating GSK3β (Manser et al., 2012, Morfini et al., 2004, Morfini et al., 2002) and by phosphorylation of NDEL1 Cdk5 inhibits dynein-mediated transport of lysosomes, autophagosomes, mitochondria, and signalling endosomes (Klinman and Holzbaur, 2015). Interestingly inactivation of Cdk5 with roscovitine rescued defective retrograde transport of TrkB vesicles in DRG neurons cultured from 90- to 100-day-old SOD1G93A transgenic mice (Klinman and Holzbaur, 2015).

#### 3.2.4 Protein aggregation

Protein misfolding and aggregation is a hallmark pathology of ALS (reviewed in Parakh and Atkin, 2016). TDP-43 aggregates are found in almost all ALS cases, including sporadic cases and most familial ALS cases. ALS patients with SOD1 mutations are a notable exception but do however exhibit aggregated mutant SOD1 in affected neurons. Other familial ALS associated mutant proteins that are prone to aggregation are TDP-43 itself, FUS, and the DPRs generated by RAN translation of the expanded G4C2 repeats in C9orf72. Furthermore, a number of familial ALS-associated proteins are known to be involved in protein quality control mechanisms, including C9orf72, valosin-containing protein (VCP), sequestosome-1/p62, ubiquilin-2, optineurin, dynactin, and TBK1 (reviewed in Webster et al., 2016b).

As discussed above, misfolded SOD1 disrupts anterograde transport by activation of p38 MAP kinase (Bosco et al., 2010). In addition, dynein has been shown to interact with ALS-mutant SOD1A4V, G85R, and G93A but not wild type SOD1 via DIC (Zhang and Zhu, 2006). Moreover dynein and ALS mutant SOD1 appeared to mostly colocalise in ALS mutant SOD1 protein aggregates in cultured motor neurons (Ligon et al., 2005) and in vivo in SOD1G93A and G85R transgenic mice (Zhang and Zhu, 2006) supporting the notion that reduced retrograde axonal transport in SOD1-related ALS may at least in part be caused by sequestration of dynein. It is not clear if any of the other misfolded proteins associated with ALS disrupt transport in a similar fashion.

In addition to TDP-43 aggregates, accumulations of neurofilaments and peripherin in axonal spheroids and motor neuron cell bodies are a pathological hallmark of ALS (reviewed in Gentil et al., 2015, Xiao et al., 2006). As described above aberrant phosphorylation of neurofilaments appears to play a major role in the dysregulation of their transport and in the formation of these pathological inclusions. It has been suggested that accumulation of neurofilaments may also affect the transport of other cargoes. Indeed, changes in neurofilament organisation due to loss of neurofilament light polypeptide (NF-L) in peripherin overexpressing cells disrupted transport of mitochondria (Perrot and Julien, 2009). Interestingly, peripherin upregulation in combination with NF-L downregulation was also found in the spinal cord of TDP-43G348C and A315T transgenic mice (Swarup et al., 2011). This may be due to TDP-43 mediated regulation of neurofilament and peripherin mRNA processing (Strong et al., 2007, Swarup et al., 2011). How neurofilament accumulations disrupt transport is not entirely resolved but may involve blockage of the axon or disruption of the microtubule network. Indeed, neurofilament depletion caused a stabilisation of microtubules in *pmn* mutant motor neurons by reducing the sequestration of Stat3/stathmin to neurofilaments (Yadav et al., 2016).

#### 3.2.5 Non-cell autonomous toxicity

Although the selective degeneration of motor neurons defines ALS, it is now clear that non-neuronal cells in the CNS such as astrocytes, microglia, and oligodendrocytes do contribute to disease. How these non-neuronal CNS cells contribute to neurodegeneration is still under debate, but may involve reduced metabolic support, release of cytokines and toxins, and glutamate excitotoxicity (reviewed in Ferraiuolo, 2014). Activated microglia-conditioned medium has been shown to induce neuritic beading in cultured motor neurons via *N-*methyl-d-aspartate (NMDA) receptor signalling (Takeuchi et al., 2005). NMDA-signalling mediated inhibition of mitochondrial complex IV and a subsequent decline in ATP levels reduced fast axonal transport and led to abnormal accumulation of tubulin, neurofilament, kinesin and dynein in the spheroids prior to the death of the motor neurons. Thus, these data suggest a link between the non-cell autonomous toxicity ALS and a downstream disruption of fast axonal transport. Similarly, expression of a mutant AR transgene solely in skeletal muscle fibres caused an androgen-dependent motor neuron degeneration and a SBMA phenotype, including defects in retrograde transport (Halievski et al., 2016).

## 4 Restoring transport as a treatment for ALS?

As discussed above, axonal transport defects are part of ALS neuropathology. Axonal transport defects are one of the earliest insults observed in ALS, arguing that they may be causative for disease. Genetic evidence showing that mutations in molecular motors and microtubules are sufficient to cause ALS and ALS-related motor neuron disorders, confirms that axonal transport defects can cause neurodegeneration. Thus, the question arises if restoring axonal transport may be of therapeutic benefit in ALS patients. The emerging insight in the molecular mechanisms underlying axonal transport defects in ALS reviewed above has allowed to devise strategies to restore transport and to begin to answer this question (Table 2).

**Table 2:** Restoring transport as a treatment for ALS. Summary of in vivo studies using the SOD1G93A transgenic mouse model, see text for details.

Target	Treatment	Effect on transport	Effect on disease	Reference
Mitochondrial docking	Syntaphilin knockout	Increases mitochondrial trafficking in DRGs	No effect	Zhu and Sheng (2011)
Dynein/retrograde transport	Cross with *Loa*	Restores retrograde endosome trafficking	Prolonged survival	Kieran et al. (2005)
Dynein/retrograde transport	Cross with *Cra1*	Not determined	Prolonged survival	Teuchert et al. (2006)
Dynein/retrograde transport	BICD2-N knockout	Not determined	Prolonged survival	Teuling et al. (2008)
Microtubules	noscapine	Normalises slow axonal transport defects	Prolonged survival	Fanara et al. (2007)
HDAC6	HDAC6 knockout	Not determined	Prolonged survival	Taes et al. (2013)
p38 MAPK kinase	Semapimod	Not determined	Prolonged survival	Dewil et al. (2007)
Calpain/Cdk5	Calpastatin overexpression	Not determined	Prolonged survival	Rao et al. (2016)
Cdk5	p35 KO	Not determined	No effect	Takahashi and Kulkarni (2004)
GSK3β	GSK-3 inhibitor VIII	Not determined	Prolonged survival	Koh et al. (2007)
GSK3β	Lithium + valproate	Not determined	Prolonged survival	Feng et al. (2008)
GSK3β	Lithium	Not determined	No effect	Gill et al. (2009)
GSK3β	Lithium	Not determined	No effect	Pizzasegola et al. (2009)

### 4.1 Restoration of mitochondrial transport

Defects in axonal transport of mitochondria are a robust finding in ALS, and because they are observed as early as at embryonic stage in ALS mouse models mitochondrial transport defects have been proposed to play an important role in disease. However, increasing axonal motility of mitochondria in SOD1G93A transgenic mice by depletion of the mitochondrial docking protein syntaphilin did not alter the course of disease in SOD1G93A transgenic mice (Zhu and Sheng, 2011). It has to be noted that Zhu et al., only verified increased axonal transport in DRG neurons which are not a target of ALS (Zhu and Sheng, 2011), but it is perhaps not surprising that restoring transport of damaged mitochondria does not affect the disease process. Indeed, the robust reduction in anterograde transport of mitochondria following ALS-associated damage may be indicative of increased clearance of mitochondria by mitophagy. Nevertheless, these findings suggest that impairment of mitochondrial transport may not be a primary cause of motor neuron degeneration and that any strategy to improve transport may need to be combined with drugs targeting mitochondrial dysfunction.

### 4.2 Restoration of endosomal trafficking

It is possible that disrupted transport of another cargo than mitochondria is essential for motor neuron survival. One such cargo may be BDNF/TrkB signalling endosomes that are retrogradely transported toward the soma (reviewed in Schmieg et al., 2014). Indeed, targeted disruption of retrograde transport causes ALS-like disease in a number of models (see above) and BDNF is known to support motor neurons in vitro and in vivo (Yan et al., 1993; reviewed in Sendtner et al., 1996).

In line with this possibility the unexpected amelioration in disease progression observed in SOD1G93A transgenic mice crossed with *Loa* mice was accompanied by a full rescue of the axonal transport of signalling endosomes (Kieran et al., 2005). The protective effect of the *Loa* mutation in dynein potentially relates to reductions in mitochondrial damage and associated axonal transport defects in SOD1G93A–*Loa*/+ mice. Indeed, the amount of mitochondria-associated mutant SOD1 protein was markedly reduced in SOD1G93A–*Loa*/+ mice and this correlated with improvements in mitochondrial respiration and membrane potential in SOD1G93A–*Loa*/+ motor neurons (El-Kadi et al., 2010). Mutant SOD1 has been shown to interact with dynein and this interaction was critical for the formation of SOD1 aggregates (Strom et al., 2008, Zhang et al., 2007). Hence a possible explanation for the restoration of endosome trafficking in SOD1G93A–*Loa*/+ mice is that reduced interaction of mutant SOD1 with *Loa* dynein restores transport of retrograde cargoes and that concomitant reductions in mutant SOD1 aggregates that are damaging to mitochondria restore mitochondrial function and possibly transport.

Overexpression of BICD2-N, which chronically impairs dynein/dynactin function, also delayed disease onset and increased life span of ‘low-copy’ SOD1G93A transgenic mice by 14% (Teuling et al., 2008). Possibly, in this case reduced transport of signalling endosomes may dampen the effects of a switch in retrograde signalling from survival to stress in SOD1G93A–*Loa*/+ transgenic mice (Perlson et al., 2009).

### 4.3 Targeting microtubules to restore transport

Microtubules are emerging as an attractive target to modulate axonal transport with several laboratories reporting beneficial effects of microtubule-binding drugs that were originally developed as antimitotic agents for the treatment of cancer (reviewed in Stanton et al., 2011). Treatment of SOD1G93A transgenic mice with noscapine, which attenuates microtubule dynamics, partially stabilised microtubules and delayed onset of disease compared to the untreated SOD1G93A transgenic mice and this effect increased when administered in combination with the anti-inflammatory PPARgamma agonist pioglitazone. Interestingly pioglitazone treatment per se also stabilised hyperdynamic microtubules (Fanara et al., 2007). Similarly, low doses of noscapine rescued peroxisome trafficking defects in HSP patient-derived olfactory neurosphere-derived cells and this correlated with a restoration of acetylated microtubules in these cells (Fan et al., 2014). In the same study, taxol, vinblastine and epothilone were reported to have a beneficial effect similar to that of noscapine (Fan et al., 2014). While both taxol and epothilone are known to stabilise microtubules, vinblastine is a microtubule-destabilising agent. However, it is likely that at the low doses used vinblastine attenuates microtubule dynamics rather than causes depolymerisation (Panda et al., 1996). If these drugs have a beneficial effect on axonal transport, axonal swellings and gait abnormalities in HSP models such as *Spast*^*∆ E7/∆E7*^ (Kasher et al., 2009) or *Sp*^∆/∆^ (Tarrade et al., 2006) mice remains to be determined, but epothilone also has shown beneficial effects in models of Alzheimer's disease (Brunden et al., 2010, Barten et al., 2012) and Parkinson's disease (Cartelli et al., 2013) and was in a clinical phase I trial for Alzheimer's disease (ClinicalTrials.gov NCT01492374).

Following a report that microtubule acetylation increased kinesin-1 mediated transport (Reed et al., 2006) several laboratories have explored the beneficial effects of acetylation enhancing drugs on axonal transport and neurodegeneration. Most of these studies have focussed on inhibition of the tubulin deacetylases HDAC6 (Hubbert et al., 2002) and SIRT2 (North et al., 2003), although the role of SIRT2 in mouse CNS remains questionable (Bobrowska et al., 2012, Taes et al., 2013). For example, HDAC6 inhibition compensated for axonal transport defects in Huntington's disease (Dompierre et al., 2007), reversed axonal loss in vivo in mutant HSPB1-induced Charcot-Marie-Tooth disease (d'Ydewalle et al., 2011), and restored axonal transport and locomotor defects in a *Drosophila* model of mutant LRRK2-associated Parkinson's disease (Godena et al., 2014) while loss of HDAC6 rendered neurons resistant to amyloid-β-mediated impairment of mitochondrial trafficking and rescued memory function in APPPS1-21 transgenic Alzheimer's disease mice (Govindarajan et al., 2013). Genetic ablation of HDAC6 in SOD1G93A transgenic mice significantly extended the survival without affecting the timing of disease onset (Taes et al., 2013). Deletion of HDAC6 increased levels of acetylated tubulin, but it is not clear if this restored the axonal transport defects reported in this mouse model (Taes et al., 2013). Although microtubule acetylation appears to be an attractive target, a possible caveat of long-term HDAC6 inhibition is that HDAC6 also plays a critical role in the clearance of aggregated proteins by autophagy (aggrephagy) (Kawaguchi et al., 2003) and has been implicated in clearance of mutant SOD1 (Lee et al., 2015b, Xia et al., 2015a, Xia et al., 2015b, Gal et al., 2013).

### 4.4 Targeting kinases to restore transport

A number of studies have targeted kinases involved in the regulation of axonal transport. Inhibition of p38 MAPK kinase protected motor neurons but only modestly prolonged survival in SOD1G93A transgenic mice (Dewil et al., 2007). Similarly, inhibition of Cdk5 by overexpression of calpastatin delayed disease onset and increased survival of SOD1G93A transgenic mice (Rao et al., 2016). Inhibition of GSK3β was found to be protective in SOD1G93A transgenic mice (Koh et al., 2007, Feng et al., 2008). Although these studies reported benefits, none analysed axonal transport so it remains to be seen if restoration of axonal transport exerts the beneficial effects observed. Moreover, since genetic ablation of the Cdk5 activator p35 did not affect the onset and progression of motor neuron disease in SOD1G93A transgenic mice (Takahashi and Kulkarni, 2004), and some studies found no effects of GSK3β inhibition (Gill et al., 2009, Pizzasegola et al., 2009) it is clear that further confirmation is required.

## 5 Conclusions

The link between axonal transport defects and ALS and other neurodegenerative diseases is very strong and over the past two decades our understanding of axonal transport defects has increased considerably. Nevertheless, there are still large gaps in our knowledge with regards to this link. For example, how do mutations in the same motors cause different neurodegenerative diseases, or why do they specifically target neurons, and often one type of neurons but not others in the same person? One explanation is the unique geometry and/or physiology of neurons renders them selectively vulnerable to insults to the transport system. As discussed above, in case of motor neurons, increased susceptibility may derive from their particular reliance on mitochondria for calcium buffering. In this scenario, mitochondrial damage combined with reduced numbers of axonal mitochondria due to defective axonal transport disrupts calcium homeostasis which in turn leads to reduced microtubule stability, impaired binding of motor proteins to the microtubules, and defective axonal transport which then exacerbates calcium dysfunction in a vicious circle. An alternative explanation may be disruption to motor neuron specific retrograde signalling pathways. However, while in ALS motor neurons exhibit the earliest signs of pathology and the highest vulnerability, there is clear evidence of pathological lesions outside the motor system. Moreover it is clear that non-neuronal cells play an important role in disease as well. Hence the apparent motor neuron specificity of ALS-associated insults such as transport deficits is relative.

Another important question is whether restoring axonal transport is sufficient to cure neurodegenerative diseases. In case of transport defects caused by mutations in the axonal transport machinery the answer to that question is probably yes and maybe gene therapy holds the future for those cases. In other cases, the answer is less clear-cut. It seems most likely that restoring axonal transport will not be a magic bullet treatment but will be of benefit in combination with other treatments targeting the diverse cellular mechanism associated with disease such as protein aggregation and mitochondrial damage. Indeed, the widespread and early nature of axonal transport defects in ALS suggests that these defects will have to be addressed if treatment is to be effective. Further efforts to standardise the measurement and analysis of axonal transport and to develop non-invasive methods to quantify axonal transport in vivo in animal models and in patients, as well as further research into the molecular mechanisms underlying axonal transport defects will be needed if we are to develop and validate treatments that target axonal transport.
